# Altered Cytokine Production in Patients with *Helicobacter pylori* Infection

**DOI:** 10.34172/mejdd.2024.398

**Published:** 2024-10-30

**Authors:** Abdollah Safikhani Mahmoodzadeh, Elham Moazamian, Seyedeh Azra Shamsdin, Gholam Abas Kaydani

**Affiliations:** ^1^Department of Microbiology, College of Sciences, Agriculture and Modern Technology, Shiraz Branch, Islamic Azad University, Shiraz, Iran; ^2^Gasteroenterohepatology Research Center, Shiraz University of Medical Sciences, Shiraz, Iran; ^3^Department of Laboratory Sciences, School of Allied Medical Sciences, Ahvaze Jundishapur University of Medical Sciences, Ahvaz, Iran

**Keywords:** Cytokines, Inflammation, Gastritis, *Helicobacter pylori*

## Abstract

**Background:**

*Helicobacter pylori* is a gram-negative pathogen. The infection caused by this pathogen may result in gastritis and can increase the risk of gastric cancer. This study investigated the relationship between *H. pylori* infection as the main risk factor for gastritis and changes in serum inflammatory cytokine levels.

**Methods:**

Blood samples from 85 patients with stomach pain, including 46 *H. pylori*-positive (Hp^+^) and 39 H. pylori-negative (Hp^-^) cases, were collected and referred to a gastroenterologist. After isolation and identification of *H. pylori*, the severity of gastritis was determined for each patient based on the histopathological findings. Finally, the serum levels of cytokines were measured using the multiplex kit and flow cytometry methods.

**Results:**

There were significant differences in the levels of interleukin-2 (IL-2), IL-4, IL-17A, IL-17F, IL-22, tumor necrosis factor-alpha (TNF-α), and interferon-gamma (IFN-γ) between the Hp^-^ and the Hp^+^ specimens (*P*≤0.05). The levels of IL-2, IL-17A, IL-17F, IL-22, TNF-α, and IFN-γ were significantly higher in patients with mild and moderate gastritis than Hp^-^ group (*P*≤0.05). In addition, IL-4 significantly increased in patients with moderate gastritis compared with Hp^-^ individuals (*P*=0.008).

**Conclusion:**

Among the inflammatory cytokines evaluated in this study, IL-17A, IL-17F, and IL-22 may play a crucial role in developing moderate gastritis in infected patients with *H. pylori*.

## Introduction

*Helicobacter pylori *is one of the most popular pathogens that infect nearly half of the population worldwide.^1^ Its prevalence is 70%-90% in developing countries and 25%-50% in the developed world.^2,3^ Gastritis is an inflammation of the stomach lining and can be acute or chronic. This disease can be caused by various reasons, such as *H. pylori* infection, long-term use of non-steroidal anti-inflammatory drugs (NSAIDs), or autoimmune reactions. Pathological diagnosis includes examining the stomach tissue for signs of inflammation, mucosal damage, inflammation, and *H. pylori* bacteria. Long-term *H. pylori *infection may lead to peptic ulcers,^4^ gastric adenocarcinoma,^5^ gastric mucosa-associated lymphoid tissue lymphoma (MALT lymphoma),^6^ and gastric cancer.^7^ Gastric cancer is the world’s fifth most common cancer, with the third cancer-induced mortality rank. About 78% of gastric cancer cases may be related to chronic infections caused by *H. pylori*.^8^

 The *H. pylori* colonization in the stomach can result in inflammatory responses recruiting the host’s innate immune cells, including macrophages, dendritic cells (DCs), lymphocytes, and neutrophils, into the gastric mucus.^9^ Upon recruitment of macrophages into the infected regions and exposure to an inflammatory stimulus, cytokines, such as interleukin (IL)- 6, IL-1, interferon-gamma (IFN-γ), and tumor necrosis factor-alpha (TNF-α) were secreted.^10^ TNF-α can activate leucocytes in* H. pylori* infections.^11^ IL-6 also contributes to the differentiation and activation of both T and B cells.^11,12^

 Innate immunity lacks memory, but adaptive immunity has immunological memory. These two immunities are related to each other after the antigen is supplied by the cells of the innate immune system to the adaptive immunity. T-helper (Th) cells are activated and differentiated, which indicates the beginning of this immune response.^13^

 T cells are required for protection against *H. pylori*, such that the immune-induced injuries of the stomach can be associated with these cells and not the B cells or antibody secretion.^13^ Generally, Th1 cells are involved in the immune responses through the production of IFN-γ, which combats several pathogens. Also, Th1 cells produce IL-2,12 along with IFN-γ.^14^ IL-4 is the cytokine responsible for differentiating T naïve cells into Th2 cells, which produce a series of cytokines, including IL-4, IL-5, IL-9, IL-10, and IL-13.^15^ Among the cytokines, TGF-β, IL-β, IL-6, and IL-23 contribute to the differentiation of T naïve cells into TH17 in humans and mice.^10^ T helper 17 (Th17) cells.^10^ IL-23 can stimulate Th17 cells to produce IL-17. Additionally, Th17 cells can produce and secrete cytokines such as IL-21, IL-22, and IL-17F.^11^ Furthermore, IL-17 can activate neutrophils and granulocytes, resulting in their chemotoxicity.^11^ Pre-inflammatory and anti-inflammatory cytokines are crucial in the inflammation among *H. pylori*-infected patients.^16^ Therefore, the clinical trend of the disease can be well predicted by measuring the level of these cytokines. This study was aimed at investigating the variations in the inflammatory cytokine levels in *H. pylori*-induced gastritis.

## Materials and Methods

###  Sample collection

 This study is a case-control study. The criteria for people to enter this study were answering the questions of the questionnaire, people with stomach pain and symptoms of indigestion, referring to gastroenterologists, and then having positive tests. Exclusion criteria: negative culture and smear, as well as pathology sample smear and negative rapid urease test (RUT) and low titer of anti-* H. pylori *antibodies in serum. This research was designed and conducted on 85 patients with gastritis and indigestion who were referred to gastroenterologists. This study was conducted in compliance with the ethical principles and consent of the participants (IR.IAU.SHIRAZ.REC.1400.003). Based on the results of the histopathological examination and rapid urease test (RUT) and positive culture, and positive titer of anti-*H. pylori* antibodies, 46 samples were *H. pylori* positive (Hp + ), and 39 samples were *H. pylori* negative (Hp^-^). The severity of gastritis was also determined for the patients. A written informed consent form was obtained from each patient. The patients’ consent statements are as follows: I give permission to use my blood sample and biopsy in the study, and my name and personal information will not be mentioned in the study. A data collection form was used to record the patients’ information, such as age and sex. All patients underwent endoscopic mucosal biopsy of the stomach. One part of the biopsy sample was used for RUT and culture to confirm the *H. pylori* infection, and the other part was immediately stored in the formalin phosphate buffer 10% to be used in histopathological examination. Additionally, 10 mL blood samples from each patient were taken, and the sera were promptly separated using centrifugation for 15 min at 3200 rpm. The sera were preserved frozen at -70 °C until used. For further confirmation of the infection of *H. pylori*, the amounts of IgG and IgA antibodies were also measured by the ELISA kit (Roche, Germany).^17^

###  Helicobacter pylori Isolation and Detection

 The stomach biopsy samples were cultured on Brucella agar plates (Merck, Germany) containing 5% sheep blood, vancomycin (10 g/mL), trimethoprim (5 g/mL), and amphotericin B (2 g/mL). Gram-staining, urease, catalase, and oxidase assays were used to identify the bacteria as *H. pylori* after the bacteria had been incubated for 3-5 days at 10% CO2, 37 °C.^18^ The bacterial suspensions were prepared from the fresh cultures, and the DNA genome was extracted using Pooyagen Azma Company Kit (Tehran, Iran) for molecular identification of *H. pylori*. The primers of *UreC* genes are presented in Table 1. The PCR process was conducted in a final volume of 25 μL containing 12.5 μL master mix 2X (Sinaclon, Iran), 1 μL of each primer, 1 μL extracted DNA, and 9.5 μL deionized water. Distilled water was used as a negative control. The PCR process was carried out using a thermocycler (BioRad, USA) with the following conditions: initial denaturation at the temperature of 94 °C for 4 minutes, then denaturation included 30 cycles at the temperature of 94 °C for 60 seconds, primer annealing at 51 °C for 30 seconds, followed by elongation at the temperature of 72 °C for 90 seconds, and final elongation at the temperature of 72 °C for 4 minutes. The reaction products were electrophoresed on a 1.5% agarose gel.

**Table 1:** The primers of the *UreC* gene

**Primer sequence**	**Gene**	**Annealing Temperature (°C)**	**Amp size (bp)**	**Reference**
5′GGATAGACGATGTGATAGG -3′	*ureC*-F	51	224 bp	^19^
5′- TTGGTTAGGGTGTAAAGC -3′	*ureC*-R			

###  Determination of the Level Of Inflammatory Cytokines by Flow Cytometry

 The serum level of the cytokines was evaluated by flow cytometry using the 13-plex LEGENDplex^TM^ Human Th Cytokine Panel Cat. No. 740001 (BioLegend, USA). First, the serum samples were diluted to 1:2 with Assay Buffer before being tested. Then, the sera were tested according to the instructions of the kit manufacturer. Finally, the contents of the well plate were transferred to fluorescence-activated cell sorting (FACS) tubes (Abcam, United Kingdom) to be read by flow cytometry (BioRad, USA).

###  Statistical Analysis

 The Kolmogorov-Smirnov test determined the normality of the data. Mann-Whitney U and independent sample *t* tests were used based on the data normality. The Chi-square test also determined relationships between qualitative variables. *P* values < 0.05 were considered statistically significant.

## Results

###  Demographic Patients 

 In this study, the demographic results of the studied subjects showed that Hp + individuals had an average age of 13 to 43, which can be said that infection occurs at a young age, and no correlation between age and sex was observed in the two groups. In the Hp + group, the severity of gastritis was mild in 11 (23.91%) and moderated in 35 (76.09%) cases. Also, a significant difference was observed in the blood indices of hemoglobin (Hb), hematocrit (Hct), red blood cell (RBC), platelets (PLT), and erythrocyte sedimentation rate (ESR) in the two groups (*P* ≤ 0.05), but there was no significant difference in the WBC (White blood cell) in the two groups. The amount of Hb, Hct, and PLT in the Hp + group decreased, and the amount of RBC, WBC, and ESR increased. The effect of *H. pylori* infection will increase RBC, WBC, and ESR (Table 2).

**Table 2:** Demographic information and some blood indices of the *H. pylori*-positive (Hp^+^) and *H. pylori*-negative (Hp^-^) groups

**Variables**	**Groups**^a^		***P *** **value**^b^
	**Hp**^-^	**Hp**^+^	
Age (y)	41.28 ± 12.65	43.83 ± 13.41	0.41
Sex (%)			0.07*
Female	22 (56.41%)	17 (36.95%)	
Male	17 (43.58%)	29 (63.05%)	
Gastritis (%)			
Mild	None	11 (23.91%)	< 0.001*
Moderate	None	35 (76.09%)	< 0.001*
Hb	13.94 ± 1.1	13.27 ± 1.48	0.022*
Hct	42.69 ± 3.42	39.93 ± 5.9	0.04*
WBC	7351 ± 1416	7756 ± 1162	0.151
RBC	4.43 ± 0.49	4.66 ± 0.42	0.022*
PLT	254.5 ± 44.35	227.6 ± 38.2	0.001*
ESR	10.21 ± 3.08	11.78 ± 3.98	0.113

Abbreviations: Hb: Hemoglobin, Hct: Hematocrit, WBC: White blood cells, RBC: Red blood cells, PLT: Platelet, ESR: Erythrocyte sedimentation rate. ^a^ Values are presented as mean ± SD or No. (%). ^b^ *P* value was considered statistically significant at level 0.05. *Significant.

###  Cytokines and IgG and IgA Antibodies

**Figure 1:**
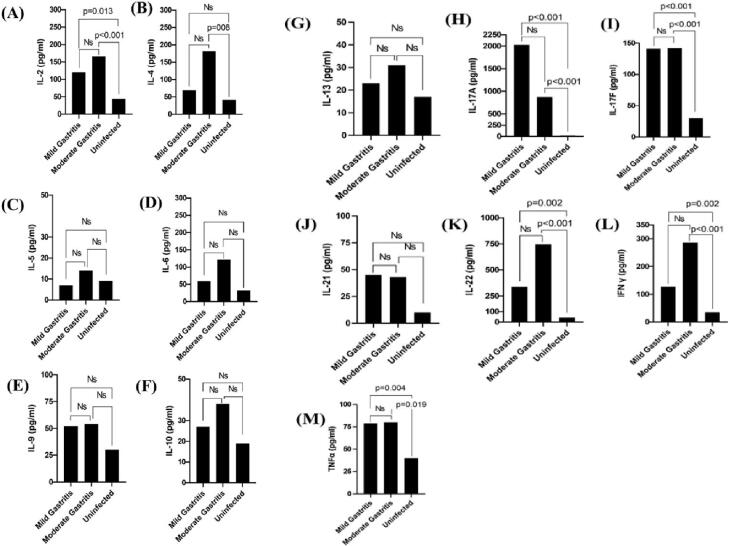
The serum levels of IL-2 (A), IL-4 (B), IL-5 (C), IL-6 (D), IL-9 (E), IL-10 (F), IL-13 (G), IL-17A (H), IL-17F (I), IL-21 (J), IL-22 (K), IFN-α (L), and TNF-γ (M) in Hp + patients with mild and moderate gastritis and uninfected (Hp^-^) group (Ns: non-significant, *P* ≤ 0.05: significant)

 Significant increases were found in IgG, IgA, IL-2, IL-4, IL-17, IL-17A, IL-17F, IL-22, TNF-α, and IFN-γ levels in the Hp^+^ group compared with the Hp^-^ group (*P* ≤ 0.05, Table 3). The amounts of IL-2, IL-4, IL-17A, IL-17F, IL-22, TNF-α, and TFN-γ were significantly different in patients with gastritis compared with Hp^-^ subjects. Accordingly, IL-2, IL-17-A, IL-17F, IL-22, TNF-α, and IFN-γ showed significant increases in patients with mild and moderate gastritis compared with the Hp^-^ subjects (Figure 1). Similarly, IL-4 showed a significant increase in patients with moderate gastritis compared with the Hp^-^ subjects (Figure 1B). Although the levels of IL-5, IL-6, IL-9, IL-10, IL-13, and IL-21 were higher in patients with gastritis than Hp^-^ group, this increase was not significant (Figure 1).

**Table 3:** The cytokines level in *Helicobacter pylori*-positive (Hp^+^) and *H. pylori*-negative (Hp^-^) groups

**Cytokines and antibodies**	**Groups**^a^		***P*** **value**^b^
	**Hp**^-^** (pg/mL)**	**Hp**^+^** (pg/mL)**	
Cytokines			
IL-2	44.82 ± 31.46	155.9 ± 212.6	< 0.001*
IL-4	41.38 ± 9.35	155.52 ± 393.4	0.005*
IL-5	9.89 ± 6.6	13.1 ± 32	0.926
IL-6	32.96 ± 27.16	107.6 ± 216.4	0.134
IL-9	30.62 ± 19.74	53.8 ± 81.8	0.114
IL-10	19.5 ± 13.37	35.7 ± 65.1	0.419
IL-13	17.42 ± 12.13	29.3 ± 38.4	0.459
IL-17A	21.34 ± 13.78	1150 ± 2447	< 0.001*
IL-17F	30.93 ± 12.47	142 ± 199.5	< 0.001*
IL-21	10.08 ± 7.65	43.9 ± 89.3	0.372
IL-22	44.43 ± 20.06	649.4 ± 2073	< 0.001*
TNF-α	40.16 ± 14.98	80 ± 95.9	0.003*
IFN-γ	35.11 ± 15.68	248.7 ± 808.8	< 0.001*
Antibodies			
IgG	9.95 ± 2.14	99.11 ± 57.2	< 0.001*
IgA	6.02 ± 2.13	74.21 ± 47.63	< 0.001*

^a^Values are presented as mean ± SD. ^b^ *P* value was considered statistically significant at level 0.05. * Significant.

## Discussion

 In this study, Hb and HCT were significantly reduced in the Hp^+^ group. These findings are similar to those reported by Kibru et al^20^ and Al Mutawa et al.^21^ However, the findings are not consistent with the studies conducted by Fraser et al^22^ in which no association was found between *H. pylori* infection and Hb/HCT count. Moreover, the results showed that RBC and PLT count significantly increased in the Hp^+^ group. These findings suggest that chronic inflammation caused by *H. pylori* infection increases the RBC and PLT. However, there was no significant increase in ESR and WBC in the Hp^+^ group. It should also be noted that ESR does not indicate inflammation. In general, the results of the hematology study of the samples show the effect of *H. pylori* infection on the hematological parameters.

 The serum levels of the inflammatory cytokines were assessed in Hp^+^ and Hp^-^ groups. Cytokine levels were different between the patients with gastritis and the Hp^-^ group. We found that *H. pylori* colonization in the stomach of Hp^+^ patients can result in an inflammatory response in recruiting the cells of the immune system, such as lymphocytes, macrophages, neutrophils, and DC, into the stomach mucus. This is in line with the previous finding that immune response and cytokines are involved in infection control and chronic inflammation.^10^

 Th1 cells produce TNF-α, IFN-γ, and IL-2.^23^ The proliferation of Th1 in *H. pylori*-infected gastric mucus involves some signals made by antigen-containing cells. The cytokines are produced in response to pathogen components, such as lipopolysaccharide, which can enhance IFN-γ secretion.^24^ Some experimental studies have indicated the dominance of Th1 response among *H. pylori*-infected persons, which may contribute to the pathogenesis of gastritis and gastric ulcers among *H. pylori*-infected cases.^25^ Studies on the gastric epithelium cell lines have shown that the production of IFN-γ can enhance MHC class II expression in the epithelium, increasing *H. pylori* attachment and probable apoptosis induction of the epithelial cells.^26^ In this study, the cytokines produced by Th1 were investigated. All three Th1-produced cytokines (i.e., IFN-γ, TNF-α, and IL-2) were significantly elevated in Hp^+^ patients. These three cytokines also showed significant increases in the patients with gastritis compared with the Hp^-^ group. Therefore, the Th1 response could be one of the underlying reasons for gastritis.

 T helper 2 cells can produce cytokines, including IL-4, IL-5, IL-10, and IL-3,^27^ and the response of these cells plays a central role in protection against infection. One previous study showed that IL-4-deficient mice had an elevated level of *H. pylori*.^28^ In this research, among the Th2-produced cytokines, only IL-4 was significantly higher in the *H. pylori*-positive cases with moderate gastritis than Hp^-^ group (*P* = 0.008). Yamaoka et al showed that IL-4 and IL-5 were not expressed in Hp^+^ and Hp^-^ groups,^29^ which is different from our results. However, the serum level of IL-4 was investigated in our study, not the gene expression.

 Similarly, this research also reported no significant difference in IL-5 serum levels. Successful colonization of *H. pylori* requires a specific balance between Th1 and Th2 cells that is determined by the host genetics.^30^ In addition, it has been revealed that the cytokines produced by Th2, especially IL-4 and IL-10, are essential for balancing and neutralizing the adverse effects of Th1.^23^ Other studies have suggested that Th2 also produces IL-6,^14^ which is a pro-inflammatory cytokine.^31^ Some studies have indicated an increased level of IL-6 mRNA in the gastric mucus of *H. pylori*-positive cases, which is directly associated with chronic gastritis.^32^ Another study has shown that the level of IL-6, along with some other cytokines, increased in patients with gastritis who had *H. pylori* infection in comparison with *H. pylori-*negative people.^33^ In this study, the IL-6 serum level showed no significant alteration between *H. pylori-*positive and *H. pylori-*negative groups. This study also addressed patients with mild or moderate gastritis, which can be considered the main reason for the differences in the previous works.^33^ Moreover, here the serum level of the cytokine was investigated, while the previous studies studied the mucosal level of the cytokines, which can be another source of difference. In one study conducted by Crabtree et al the serum level of IL-6 was greater in patients with gastric cancer than in those with benign *H. pylori*-induced gastric lesions.^34^ However, the type of sample (cancer samples) was different from our study.

 T helper 17 cells can secrete IL-17A, IL-17F, IL-21, and IL-22.^35^ It has been well documented that IL-17 is vital in the pathogenesis of some chronic inflammatory diseases. For instance, IL-17 was observed in rheumatoid arthritis (RA) and osteoarthritis synovial fluids.^36^ In our study, IL-17A showed a significant increase in Hp^+^ patients, which is in line with the results reported by Arachchi et al in 2017.^37^ Increased expression of the *IL-17* gene (compared to Hp^-^) has been reported in the *H. pylori*-infected biopsies.^38^ Moreover, high levels of IL-17 in the gastric mucus have been reported at the infection site. It is proposed that IL-7 cytokine has a determining role in neutrophil recruitment to the *H. pylori*-infected gastric mucus and stimulates the fibroblasts to produce matrix metalloproteinases, leading to further gastric mucus damage.^39^ In addition, IFN-γ regulates Th17 cell induction.^36^ Therefore, Th1 cells that produce IFN-γ are also involved in Th17 regulation. In the present study, IFN-γ cytokine was significantly higher in the *H. pylori*-infected cases. Furthermore, Serelli-Lee et al reported that even after the eradication of *H. pylori*, the remaining Th17 cells and, hence, their induced IL-7A response may be involved in gastric cancer from the initial stages of *H. pylori* infection.^39^ Also, it is reported that IL-17A and IL-17F possess highly similar homology; however, IL-17F may possess a more prominent role in the intrinsic response than IL-17A.^40^ In our study, the amount of IL-17F was also increased in Hp^+^ patients.

 IL-21 is an autocrine cytokine causing Th17 enhancement. It has various effects on the inflammation trend, such that it plays a direct role in the inflammatory damage of tissues and destruction of the intestine of patients with inflammatory bowel disease.^41^ Our study showed no significant difference in IL-21 between groups, which is the same as the results obtained by Shamsdin et al in 2015.^41^

 In our study, IL-22 had a significant increase in mild (*P* = 0.002) and moderate (*P* < 0.001) gastritis compared with the Hp^-^ group. In another study by Shamsdin et al,^41^ they showed that IL-22 significantly increased in patients with moderate gastritis, while it was not significant in patients with mild gastritis. However, some other studies reported no increase in IL-22 among patients with gastritis.^41^

## Conclusion

 Among the inflammatory cytokines evaluated in this study, IL-17A, IL-17F, and IL-22 can play a crucial role in developing gastritis, especially moderate gastritis, in infected patients with *H. pylori*.
